# A prospective cohort study of racial/ethnic variation in the association between change in cystatin C and dietary quality in older Americans

**DOI:** 10.1017/S0007114522001040

**Published:** 2023-01-28

**Authors:** Nicholas J. Bishop, Jie Zhu

**Affiliations:** 1 Human Development and Family Sciences Program, School of Family and Consumer Sciences, Texas State University, San Marcos, TX 78666, USA; 2 Nutrition and Foods Program, School of Family and Consumer Sciences, Texas State University, San Marcos, TX 78666, USA

**Keywords:** Chronic kidney disease, Energy-adjusted AHEI-2010, Cystatin C, Ageing, Dietary quality

## Abstract

Using a sample of US adults aged 65 years and older, we examined the role of dietary quality in cystatin C change over 4 years and whether this association varied by race/ethnicity. The Health and Retirement Study provided observations with biomarkers collected in 2012 and 2016, participant attributes measured in 2012, and dietary intake assessed in 2013. The sample was restricted to respondents who were non-Hispanic/Latino White (*n* 789), non-Hispanic/Latino Black (*n* 108) or Hispanic/Latino (*n* 61). Serum cystatin C was constructed to be equivalent to the 1999–2002 National Health and Nutrition Examination Survey (NHANES) scale. Dietary intake was assessed by a semi-quantitative FFQ with diet quality measured using an energy-adjusted form of the Alternative Healthy Eating Index-2010 (AHEI-2010). Statistical analyses were conducted using autoregressive linear modelling adjusting for covariates and complex sampling design. Cystatin C slightly increased from 1·2 mg/l to 1·3 mg/l over the observational period. Greater energy-adjusted AHEI-2010 scores were associated with slower increase in cystatin C from 2012 to 2016. Among respondents reporting moderately low to low dietary quality, Hispanic/Latinos had significantly slower increases in cystatin C than their non-Hispanic/Latino White counterparts. Our results speak to the importance of considering racial/ethnic determinants of dietary intake and subsequent changes in health in ageing populations. Further work is needed to address measurement issues including further validation of dietary intake questionnaires in diverse samples of older adults.

Chronic kidney disease (CKD) poses a global public health problem^(1–3)^ as it is a primary risk factor for cardiovascular morbidity and mortality^(4,5)^ and is accelerated by common chronic conditions including hypertension, diabetes, and obesity^(6)^. CKD affects 11–13 % of adults worldwide^(2)^, about 15 % adults in the USA, and 38 % of US adults aged 65 years and older^(7)^. The risk of CKD varies by race/ethnicity with non-Hispanic/Latino Black adults being at greater risk of decline in kidney function than non-Hispanic/Latino White and Hispanic/Latino adults^(8–10)^. The incidence of end-stage renal disease is also higher among Hispanic/Latinos than non-Hispanic/Latino Whites^(11–13)^. Moreover, it has been reported that from 2019 to 2040, non-Hispanic/Latino White adults aged 65 years and older are projected to increase 29 %, while non-Hispanic/Latino Black and Hispanic/Latinos in the same age group are projected to increase 80 % and 161 %, respectively^(14)^. Thus, it is critical to identify potentially modifiable risk factors, such as diet, in the progression of CKD among older adults from diverse racial/ethnic backgrounds. However, limited research has examined the role of dietary quality on kidney function decline, especially considering racial/ethnic variation in CKD risk among ageing adults.

Cystatin C is an indicator of kidney function, identified as a potentially superior biomarker than creatinine for the estimation of glomerular filtration rate (GFR)^(15,16)^, with benefits including being unaffected by muscle mass^(17)^ and changes in dietary protein intake^(18)^, and enhanced ability to detect small reductions in GFR^(19)^. In addition to being a promising biomarker of early-stage CKD, serum cystatin C has been identified as a predictor of CKD-related complications^(17)^ and an independent risk factor for CVD^(15,19–21)^ and all-cause and cardiovascular mortality among older adults without renal impairment or with CKD^(22,23)^.

Dietary modifications are a primary tool for the management of CKD and end-stage renal disease, and studies examining the role of dietary patterns in the progression of CKD are better equipped to capture the complex interactions between nutrients that occur in everyday dietary intake compared with studies of individual nutrients^(24)^. Greater dietary quality measured using several dietary indices have been associated with decreased risk of CKD and renal health outcomes^(24–27)^, and the role of dietary quality in CKD appears to vary by race/ethnicity. Greater adherence to healthy dietary patterns during middle age was negatively associated with decline in kidney function among US Hispanic/Latinos^(28)^ and risk of CKD among US non-Hispanic/Latino White and Black adults^(29)^. Existing research indicates the importance of dietary quality in kidney function and incidence of CKD and related outcomes, but we are unaware of work examining the role of dietary quality in changes in cystatin C as an indicator of early-stage CKD in community-dwelling older Americans. In addition, it is crucial to consider potential racial/ethnic variation in the role of dietary quality in changes in kidney function, given racial/ethnic variation in CKD incidence and prevalence, and the shifting demographic makeup of our ageing population. To these ends, we examined the association between change in cystatin C over 4 years and dietary quality using an energy-adjusted version of the Alternative Healthy Eating Index-2010 (AHEI-2010), a measurement of food and nutrient intake predictive of chronic disease risk^(30)^ and further explored whether this association varied by race/ethnicity.

## Materials and methods

### Study population

Observations were drawn from the Health and Retirement Study (HRS) and Health Care and Nutrition Study (HCNS). The HRS is a biennial panel survey of older Americans funded by the US National Institute on Aging and the US Social Security Administration^(31)^. The HCNS is a mail-out supplement of the HRS measuring food consumption in a subsample of HRS respondents^(32)^. Self-report measures were drawn from the cleaned RAND HRS data file^(33)^ and longitudinal measurement of dried blood spots (DBS) were taken from the 2012 and 2016 HRS. In 2016, a Venous Blood Study (VBS) was conducted on a subsample of HRS respondents and measured a variety of biomarkers not available in the HRS DBS data, with a 65 % final completion rate among eligible cases^(34)^. The HRS protocol includes a spoken confidentiality statement, oral or implied consent at first contact, and written informed consent at each interview. The University of Michigan’s Institutional Review Board approved the HRS and HCNS protocol^(35)^. Response rates for the 2012 HRS and 2013 HCNS were 89·1 %^(36)^ and 65 %^(32)^, respectively, and the HRS and HCNS were conducted in English or Spanish, based on the preference of the respondent.

The initial dataset included 2766 respondents who had valid cystatin C measurement in 2012. Participants were excluded from analyses if they were under the age of 65 years (*n* 1259), had missing information on cystatin C in 2016 (*n* 471), daily energy intakes fell outside the common allowable range of 500 to 3500 kcal/d (2092 to 14644 kj/d) for women and 800 to 4000 kcal/d (3347 to 16736 kj/d) for men (*n* 56)^(37)^. Respondents who reported race/ethnicity other than non-Hispanic/Latino White, non-Hispanic/Latino Black, or Hispanic/Latino (*n* 22) were also excluded. These restrictions resulted in an analytic sample of 958 respondents, which included 63·6 % of the age-eligible sample. Of the 958 respondents included in primary analyses, 733 were also measured in the 2016 HRS VBS. A flow chart depicting participant selection in our study is provided in Supplementary Fig. 1.

### Biomarker assessment

DBS were collected during an enhanced face-to-face interview requiring special informed consent, with consent rates of 87 % in 2012^(38)^ and 86·7 % in 2016^(39)^. The DBS samples were assayed for five biomarkers including cystatin C at the University of Washington Department of Medicine Dried Blood Spot Laboratory^(38)^. Cystatin C (mg/l) was assayed by ELISA using the BioVendor Human cystatin C ELISA kit (mg/l)^(40)^. We chose to analyse cystatin C in its original metric rather than transform to estimated glomerular filtration rate (eGFR) as the majority of eGFR equations include both cystatin C and serum creatinine^(16)^, and creatinine was not available for analysis in the 2012 HRS DBS.

Other biomarkers available in the 2012 HRS DBS were included as covariates in multivariate analyses. Glycosylated haemoglobin (HbA1c; %) was assayed using a Bio-Rad Laboratories Variant II High-Pressure Liquid Chromatography System^(41)^. Percentages of HbA1c were categorised to represent clinical thresholds of normal (<5·7 %; reference), pre-diabetic (5·7 %–6·4 %), and diabetic (≥ 6·5 %)^(42)^. Total cholesterol (mg/dl) was measured using a microtiter plate assay, and the C-reactive protein (ug/ml) assay was performed using ELISA^(38)^. Total cholesterol was categorised to represent desirable (<200 mg/dl; reference), borderline high (200–239 mg/dl), and high (≥ 240 mg/dl)^(43)^. To standardise biomarker values across differing assays and laboratories, values equivalent to the National Health and Nutrition Examination Survey (NHANES) were created by assuming the distributions of HRS DBS assays were similar to values reported in NHANES. Values for both HRS DBS and NHANES assays were calculated for each percentile, then transformed into the NHANES scale^(38)^. The 2012 and 2016 measures of cystatin C were constructed to be equivalent to the 1999–2002 NHANES scale, HbA1c and total cholesterol were compared with NHANES 2013–2016 values, and NHANES 2015–2016 was referenced for C-reactive protein^(38)^. The HRS recommends use of the equivalent assays for analytic purposes, and further description of the calculation of equivalent values is available^(38)^.

In the 2016 VBS, 50·5 ml of blood was collected in six tubes by a standard phlebotomy service with tube processing done within 48 h of collection. Available assays germane to our study of cystatin C include creatinine (mg/dl), blood protein (g/dl), albumin (g/dl), alanine aminotransferase (u/l), aspartate aminotransferase (u/l), bilirubin (total, mg/dl), blood urea nitrogen (BUN; mg/dl), LDL cholesterol (mg/dl), and TAG (mg/dl). Complete description of laboratory equipment, assays, and quality control procedures are available^(34)^.

### Dietary intake

An energy-adjusted version of the AHEI-2010 was examined as a measure of dietary quality. A modified Harvard FFQ^(32,44)^ was used to measure dietary intake in the HCNS, and calculated energy and nutrient totals were based on standard nutrient tables^(45)^. This FFQ was not validated in the HCNS sample, but a validation study of a similar FFQ demonstrated an average energy-adjusted Pearson’s correlation of .46 for comparative validity between nutrient intake on the FFQ and multiple 24-h dietary recall interviews in a sample of older biracial adults^(46)^. Respondents used common portion sizes to report average total consumption of each food item over the past 12 months, which were converted to average servings per day. To adjust for extraneous variation in estimates of dietary intake related to total energy intake, we used an energy-adjusted version of the AHEI-2010. Residuals were estimated by regressing the original AHEI-2010 components on total energy intake, producing residuals that reflect the difference between intake of the specific AHEI-2010 component and what would be expected given the respondent’s total energy intake^(47)^. We also adjusted for energy intake in our primary statistical models to account for potential effects of energy intake in addition to diet composition as represented by the energy-adjusted AHEI-2010 scores. Deciles of residuals for each AHEI-2010 component were calculated with a range from 0 to 9, resulting in a summed energy-adjusted AHEI-2010 score ranging from 0 to 90, with higher scores indicating better dietary quality.

Racial/ethnic variation in the separate components of the AHEI-2010 (non-energy-adjusted), macronutrient density, and micronutrients relevant to CKD were examined to support primary analyses. AHEI-2010 components included daily servings of vegetables, fruit, whole grain, sugar-sweetened beverages, nuts and legumes, red/processed meats, and alcohol, % daily energy from trans-fat, mg/d of long-chain (*n*-3) fatty acids (EPA + DHA), and % daily energy from PUFA^(30)^. Macronutrient density for daily carbohydrate, protein, and fat intake was calculated as the estimated percent of daily energy content coming from each macronutrient source (% kcal). Intake of specific macronutrients and micronutrients related to health and kidney function were examined to support primary analyses and can be found in Supplementary Table 2. Please note that energy-adjusted AHEI-2010 was used in the primary analyses to produce statistical estimates adjusted for potential measurement error in self reports of dietary intake, while the separate components of non-energy-adjusted AHEI-2010 and macronutrient density were only included in supplementary analyses to provide readers with additional indicators of racial/ethnic variation in dietary intake.

### Race/ethnicity, interview language and nativity

Respondents’ race/ethnicity was self-reported. Respondents who reported only a single race (i.e. non-Hispanic/Latino White or non-Hispanic/Latino Black/African American) or Hispanic/Latino ethnicity were identified as that race/ethnicity. When a respondent reported both one of the possible racial categories and Hispanic/Latino ethnicity, the individual was identified as Hispanic/Latino. For brevity, we refer to non-Hispanic/Latino White and non-Hispanic/Latino Black/African American respondents as White or Black respondents throughout the remainder of the manuscript. To further adjust models for covariates related to race/ethnicity, an indicator of whether the core 2012 HRS interview was conducted in Spanish (Spanish/English) was included to adjust for potential language issues related to completion of the HRS and HCNS, and an indicator of whether the respondent was born in the USA (born in the USA/not born in the USA) was included as a measure of nativity.

### Covariates

Analyses adjusted for relevant covariates including age, sex (female/male), marital status (partnered or married/single, divorced, or widowed) and retirement status (retired/not retired). Education (<12 years of education, 12 years of education, >12 years of education), household income and household assets accounted for socio-economic resources, and an indicator of food security status was included due to potential associations between dietary quality and food insecurity among older adults^(48)^. Food insecurity was assessed using the six-item short form of the US Household Food Security Survey Module^(49)^ included in the HCNS, with missing responses to any of the food security items resulting in a missing food security score (food-insecure/food-secure). Covariates representing health behaviours included BMI (underweight (<18·5 kg/m^2^), normal weight (between 18·5 kg/m^2^ and 25 kg/m^2^), overweight (between 25 kg/m^2^ and 30 kg/m^2^), and obese (≥ 30 kg/m^2^)), vigorous physical activity (participation in activities such as sports, heavy housework, or a job that involves physical labour; no vigorous physical activity, vigorous physical activity <1 time per week, vigorous physical activity >1 time per week) and smoking status (current smoker/non-smoker). Multimorbidity was measured as a sum of seven items asking whether the respondent had been diagnosed with cancer (any malignant tumour excluding skin cancer), lung disease (such as chronic bronchitis or emphysema excluding asthma), heart disease (including myocardial infraction, CHD, angina, congestive heart failure or other heart problems), stroke, psychiatric problems or arthritis, as well as an indicator of depression. Depressive symptoms were identified using the Center for Epidemiological Studies Depression (CES-D) scale adapted in the HRS with responses to the eight depressive symptoms coded in a yes/no format with a cut-off of ≥ 4 indicating high depressive symptomatology^(50)^. Respondents with missing information on any of the seven multimorbidity items were coded as missing. Also, an indicator of self-reported diabetes-related kidney trouble or protein in urine was included as a covariate. Blood pressure was measured during in-person interviews in 2012 using an automated device validated against manual measurement^(51,52)^. Three separate measurements of systolic and diastolic blood pressure were averaged, then classified (normal: <120 systolic and 80 diastolic; elevated: 120–129 systolic and <80 diastolic; stage 1 hypertension: 130–139 systolic or 80–89 diastolic; stage 2 hypertension: ≥ 140 systolic or ≥ 90 diastolic; hypertensive crisis: >180 systolic and/or >120 diastolic; hypotension: ≤ 90 systolic or ≤ 60 diastolic)^(53)^. Finally, estimated daily energy intake, measured as average daily total kilocalorie (kcal) intake, was included to adjust for overall energy intake.

### Statistical analyses

The primary aim of our secondary observational analysis was to estimate the association between change in cystatin C and dietary quality and identify whether this association varied by race/ethnicity. Change in cystatin C from 2012 to 2016 was modelled by regressing cystatin C measured in 2016 on cystatin C measured in 2012, described as the regressor variable method of analysing change scores^(54)^, or a first-order autoregressive model. Model 1 estimated the change in cystatin C from 2012 to 2016 adjusted for all independent variables, and model 2 included interaction terms between energy-adjusted AHEI-2010 and race/ethnicity dummy variables, using White older respondents as the reference category. Supplementary models were used to identify whether there were pre-existing racial/ethnic differences in the association between energy-adjusted AHEI-2010 and baseline (2012) cystatin C.

Linear regression models were completed using maximum likelihood estimation with robust standard errors, providing treatment of missing data, adjustment of standard errors for non-normality, and adjustment for correlations between independent variables. Sample weights from the 2012 DBS and indicators of household clustering and geographic stratum from the HRS were used to adjust analyses for differential probabilities of sample selection related to the multi-stage, area-clustered, and stratified sample design of these studies. Mplus 8.4^(55)^ was used to estimate the linear regression models and produce Johnson–Neyman plots to identify regions of the energy-adjusted AHEI-2010 where the association between change in cystatin C and dietary quality significantly varied by race/ethnicity^(56)^. All continuous variables included as covariates were grand-mean-centred. To account for the strong right-skewness of C-reactive protein, household income, and household assets, these measures were log-transformed before inclusion as covariates in statistical models. In the linear regression models, energy-adjusted AHEI-2010 was rescaled by dividing the original energy-adjusted AHEI-2010 by a value of 10 to prevent potential estimation errors related to large variances. Multi-collinearity was not problematic with variance inflation factors for all predictor variables <4^(57)^. The *α* level of statistical significance used in our study was *P* < 0·05. As our study was a secondary analysis of observational data based on a large sample, formal power analysis was not required based on journal guidance.

SAS 9.4^(58)^ was used to analyse weighted descriptive data and test bivariate associations adjusted for complex sampling design. Overall differences in continuous measures were compared across racial/ethnic groups using ANOVA, with least squares mean differences used for bivariate follow-up. Differences in categorical measures across racial/ethnic group were tested with the Rao–Scott χ^2^ test. Significant overall χ^2^ tests were partitioned into 2 × 2 contingency tables with Rao–Scott χ^2^ tests used to test significance, with odds ratios (OR) used to identify the direction of association. The significance level for each follow-up test was adjusted using the Bonferroni correction.

## Results

### Descriptive statistics

Table 1 presents descriptive statistics for cystatin C, energy-adjusted AHEI-2010 and all covariates. Mean cystatin C was 1·2 mg/l (sd = 0·4) in 2012 and 1·3 mg/l (sd = 0·5) in 2016. Though the overall test of differences in cystatin C in 2012 by racial/ethnic group was statistically significant, bivariate comparisons did not suggest racial/ethnic differences in cystatin C at baseline. The average energy-adjusted AHEI-2010 score for the sample was 49·7 (sd = 13·1), which did not significantly differ by race/ethnicity. The mean age of the sample was 73·1 years (sd = 5·9). Average daily energy intake was 1753 kcal (7334.6 kj; sd = 632·1), which did not significantly differ by race/ethnicity. Only 37 % of Hispanic/Latinos were born in the USA, and of the 61 Hispanic/Latino respondents, 34 (57·6 % based on weighted frequency) completed the core 2012 HRS in Spanish. Black respondents were significantly more likely to report diabetes-related kidney issues than White respondents and appeared to have significantly greater C-reactive protein levels and diabetes risk based on HbA1C than White or Hispanic/Latino respondents.

**Table 1.** Weighted descriptive statistics for cystatin C, energy-adjusted AHEI-2010, and covariates, Health and Retirement Study (2012, 2016), Health Care and Nutrition Study (2013) (Numbers and percentages, mean values, and standard deviations)

	Total sample			Non-Hispanic/Latino White				Non-Hispanic/Latino Black				Hispanic/Latino					
Primary measures	*n*	Mean	sd	*n*	Mean		sd	*n*	Mean		sd	*n*	Mean		sd	*F^§^*	*P*
Cystatin C (mg/l), 2012	958	1·2	0·4	789	1·2	^a,b^	0·4	108	1·3	^a,c^	0·5	61	1·2	^b,c^	0·6	3·04	0·048
Cystatin C (mg/l), 2016	958	1·3	0·5	789	1·2		0·5	108	1·3		0·5	61	1·3		0·5	1·1	0·332
Energy-adjusted AHEI-2010^†^, 2013	958	49·7	13·1	789	49·7		13·2	108	47·2		12·8	61	52·2		12·4	2·08	0·126
Continuous covariates																	
Age (years)	958	73·1	5·9	789	73·2		6·0	108	71·7		5·2	61	73·2		6·2	1·68	0·188
Household income ($1000)^‡^	958	79·0	125·7	789	79·0		136·5	108	41·4		42·1	61	25·1		25·8	41·67	<0·001
Household assets ($1000)^‡^	958	712·0	1218·9	789	712·0		1317·1	108	177·0	^a^	288·9	61	182·8	^a^	366·2	30·65	<0·001
Multimorbidity	925	1·7	1·2	761	1·7		1·2	106	1·8		1·2	58	1·7		1·3	0·64	0·526
2012 C-reactive protein^‡^	956	3·5	6·8	788	3·4	^a^	7·1	108	5·3	^a,b^	5·5	60	2·6	^b,c^	3·9	11·38	<0·001
Energy intake (Kcal)	958	1753·1	632·1	789	1754·4		616·5	108	1682·1		738·4	61	1837·1		638·3	0·90	0·426
Categorical covariates	*n*	%		*n*	%			*n*	%			*n*	%			*χ* ^*2|*^	*P*
Sex/gender																	
Male	357	42·1		322	43·5			31	30·8			24	33·6			6·0	0·049
Female	581	57·9		467	56·5		^*^	77	69·2	^*^		37	66·4				
Marital/partnership status																	
Single/divorced/widowed	332	33·8		238	30·5			67	61·4			27	54·8			31·5	<0·001
Married/partnered	626	66·2		551	69·5		^*^	41	38·7	–		34	45·2		–		
Retirement status																	
Not retired	281	31·3		220	30·9			32	33·9			19	35·3			0·5	0·782
Retired	687	68·7		569	69·1			76	66·1			42	64·7				
Interview language																	
English	923	97·3		788	100·0			108	100·0			27	42·4			–	**–**
Spanish	35	2·7		1	0·0		–	0	0·0			34	57·6		^*^		
Nativity status																	
Born in the USA	876	8·2		39	5·7			5	3·6			37	62·6			–	–
Not born in the USA	81	91·8		749	94·2		^*^	103	96·4			24	37·4		–		
Missing	1	0·1		1	0·1			0	0·0			0	0·0				
Education																	
<High school degree	163	13·6		97	10·3		–	29	24·7	^*^		37	58·8			97·0	<0·001
High school degree	354	36·6		303	37·4			39	40·1			12	16·3		–		
>High school degree	441	49·9		389	52·3		^*^	40	35·2	–		12	24·9		–		
Food security status																	
Food-secure	807	85·9		693	89·0		^*^	78	69·4	–		36	51·3		–	54·4	<0·001
Food-insecure	87	7·8		53	6·1		–	19	16·8	^*^		15	25·3		^*^		
Missing	64	6·3		43	4·8		–	11	13·8	^*^		10	23·4		^*^		
BMI																	
Underweight	18	2·2		16	2·0			0	0·0			2	8·8		^*^	**–**	**–**
Normal	366	25·4		219	26·7		^*^	20	17·0			10	15·2				
Overweight	325	37·5		301	37·5			37	33·6			28	44·0				
Obese	508	34·9		253	33·9			51	49·5	^*^		21	32·1				
Vigorous physical activity																	
None	508	50·8		411	50·1			64	60·4			33	49·3			3·2	0·524
Some	214	23·4		179	24·1			22	17·4			13	19·4				
Regular	234	25·7		198	25·7			22	22·2			14	30·4				
Smoking status																	
Non-smoker	890	92·6		735	92·7			97	88·5			58	90·4			1·0	0·597
Current smoker	63	7·4		50	7·0			11	11·5			2	8·3				
Missing	5	0·4		4	0·3			0	0·0			1	1·4				
Diabetic kidney problems																	
No	936	97·8		774	98·2		–	103	92·0			59	98·0			23·6	<0·001
Yes	14	1·1		9	0·7			4	6·6	^*^		1	1·1				
Missing	8	1·1		6	1·1			1	1·4			1	1·0				
Blood pressure																	
Normal	233	24·6		195	24·7			21	20·8			17	28·3			**–**	**–**
Elevated	164	17·6		137	18·2			18	14·8			9	11·1				
Stage 1 hypertensive	214	23·6		176	24·0		^*^	27	24·4			11	16·3				
Stage 2 hypertensive	303	29·6		243	28·3			38	37·0			22	42·2				
Hypertensive crisis	15	1·9		13	2·0			2	2·0			0	0·0				
Hypotensive	29	2·7		25	2·9			2	1·0			2	2·1				
Total cholesterol																	
Normal	605	61·7		494	61·4			68	60·2			43	69·4			1·7	0·801
Borderline high	255	28·2		214	28·4			29	27·8			12	24·3				
High	98	10·2		81	10·2			11	12·0			6	6·4				
HbA1c																	
Normal	497	56·1		434	57·9			30	31·8			33	57·9			33·2	<0·001
Pre-diabetic	283	28·7		238	28·7		^*^	32	30·0			13	25·8				
Diabetic	178	15·3		117	13·4		–	46	38·2	^*^		15	16·3				

† AHEI-2010, Alternative Healthy Eating Index-2010.

‡ Means and standard deviations for non-log-transformed measure, and statistical tests for log-transformed measure.

§ Overall differences in continuous measures were compared using ANOVA, with least squares mean differences used for bivariate follow-up. The significance level for each follow-up test was adjusted using the Bonferroni correction.

^|^Differences in categorical measures were tested with the Rao–Scott χ^2^ test. Significant overall χ^2^ tests were partitioned into 2 × 2 contingency tables with Rao–Scott χ^2^ tests used to test significance, with OR identifying the direction of association. For categorical variables, overall χ2 not estimated for variables including zero counts in race/ethnicity two-way table. The significance level for each follow-up test was adjusted using the Bonferroni correction.

a,b,c Matching superscript letters denote non-significant pairwise comparison.

* Significantly greater observed *v*. expected count, - significantly fewer observed *v*. expected count in cell.

### Primary regression models

Table 2 presents results from the regression models estimating change in cystatin C from 2012 to 2016. Model 1 estimated the direct association between change in cystatin C from 2012 to 2016, energy-adjusted AHEI-2010, race/ethnicity, and all covariates. Energy-adjusted AHEI-2010 was negatively associated with change in cystatin C, with a 10-unit increase in energy-adjusted AHEI-2010 being associated with a .03-unit decrease in cystatin C change from 2012 to 2016 (*b* = –0·03, 95 % CI = –0·05, -0·01). Age was positively associated with change in cystatin C, and older adults who completed the 2012 core HRS interview in Spanish or who reported being born in the USA tended to have more rapid increase in cystatin C than their counterparts. Obese older adults had a .13-unit greater increase in cystatin C than those with a normal BMI (*b* = 0·13, 95 % CI = 0·06, 0·20), and older adults reporting diabetes-related kidney problems had a .69-unit greater increase in cystatin C than those not reporting such problems (*b* = 0·69, 95 % CI = 0·15, 1·21). Older adults identified as either stage 2 hypertensive or experiencing hypertensive crisis also had greater increases in cystatin C than those with normal blood pressure.

**Table 2.** Weighted linear regression estimates of the association between change in cystatin C (2012–2016), energy-adjusted AHEI-2010, and race/ethnicity; Health and Retirement Study (2012–2016) and Health Care and Nutrition Study (2013)

	Model 1^†^		Model 2^‡^	
	Direct effect change model		Non-Hispanic/Latino White reference	
Primary measures	*b^§^*	95 % CI	*b^§^*	95 % CI
Cystatin C, 2012	0·57	0·49, 0·66	0·57	0·49, 0·66
Energy-adjusted AHEI-2010 (DIV10)^||^	–0·03	–0·05, –0·01	–0·04	–0·06, –0·01
Race/ethnicity				
Non-Hispanic/Latino White (ref)				
Non-Hispanic/Latino Black	–0·09	–0·21, 0·03	–0·08	–0·20, 0·04
Hispanic/Latino	–0·05	–0·17, 0·06	–0·05	–0·17, 0·07
Interaction terms				
Energy-adjusted AHEI-2010 X non-Hispanic/Latino White (ref)		–	–	–
Energy-adjusted AHEI-2010 X Non-Hispanic/Latino Black			0·03	–0·05, 0·12
Energy-adjusted AHEI-2010 X Hispanic/Latino			0·07	0·02, 0·13
Covariates				
Age	0·01	0·01, 0·02	0·01	0·01, 0·02
Female	0·00	–0·06, 0·06	0·00	–0·06, 0·06
Married/partnered	0·04	–0·02, 0·10	0·04	–0·02, 0·10
Retired	–0·04	–0·11, 0·03	–0·04	–0·11, 0·03
Spanish interview	0·15	0·00, 0·31	0·12	–0·04, 0·28
Native born	0·07	0·01, 0·14	0·08	0·01, 0·15
Education				
<High school degree	0·01	–0·09, 0·11	0·02	–0·08, 0·12
High school degree (reference)				
>High school degree	–0·03	–0·09, 0·04	–0·03	–0·09, 0·04
Household assets, log	–0·01	–0·01, 0·00	–0·01	–0·01, 0·00
Household income, log	0·01	–0·03, 0·05	0·01	–0·03, 0·05
Food-insecure	–0·04	–0·16, 0·09	–0·03	–0·16, 0·09
BMI				
Underweight	0·14	–0·04, 0·32	0·15	–0·03, 0·33
Normal (reference)				
Overweight	0·04	–0·02, 0·10	0·04	–0·02, 0·10
Obese	0·13	0·06, 0·20	0·13	0·06, 0·20
Vigorous physical activity				
None (reference)				
Moderate	0·00	–0·07, 0·06	0·00	–0·07, 0·06
Regular	–0·01	–0·07, 0·06	–0·01	–0·07, 0·06
Current smoker	0·09	–0·01, 0·19	0·09	–0·01, 0·19
Sum chronic conditions	0·01	–0·02, 0·03	0·01	–0·02, 0·03
Diabetic kidney problems	0·69	0·16, 1·21	0·68	0·15, 1·21
Blood pressure				
Normal (reference)				
Elevated	0·01	–0·06, 0·08	0·01	–0·06, 0·08
Stage 1 hypertensive	0·02	–0·06, 0·09	0·02	–0·06, 0·09
Stage 2 hypertensive	0·13	0·05, 0·20	0·12	0·05, 0·20
Hypertensive crisis	0·20	0·00, 0·40	0·20	–0·01, 0·40
Hypotensive	–0·06	–0·20,·08	–0·06	–0·20, 0·08
HbA1c				
Normal (reference)				
Pre-diabetic	0·02	–0·04, 0·07	0·02	–0·04, 0·07
Diabetic	0·05	–0·03, 0·14	0·05	–0·03, 0·14
Cholesterol				
Desirable (reference)				
Borderline high	–0·02	–0·08, 0·04	–0·02	–0·08, 0·05
High	–0·05	–0·14, 0·05	–0·05	–0·14, 0·05
C-reactive protein (log)	0·02	–0·02, 0·05	0·02	–0·02, 0·05
Energy intake (kcal/100)	0·00	0·00, 0·01	0·00	0·00, 0·01
R-squared	0·45		0·46	

† Model 1, cystatin C change (2012–2016) direct effect model.;

‡ Model 2, cystatin C change (2012–2016) interaction effect model, non-Hispanic/Latino White reference.

§ b, regression estimate.

|| Energy-adjusted AHEI-2010 (DIV 10), Energy-adjusted Alternative Healthy Eating Index-2010, divided by 10.

Model 2 added interaction terms between energy-adjusted AHEI-2010 and the indicator variables for race/ethnicity to the previous model. The interaction between energy-adjusted AHEI-2010 and the indicator variable for Black respondents was not significant (*b* = 0·03, 95 % CI = –0·05, 0·12), but the interaction between energy-adjusted AHEI-2010 and the indicator of Hispanic/Latino ethnicity was significant (*b* = 0·07, 95 % CI = 0·02, 0·13), suggesting the association between energy-adjusted AHEI-2010 and change in cystatin C was significantly different for White and Hispanic/Latino respondents.

To identify whether the association between change in cystatin C and energy-adjusted AHEI-2010 was significantly different for White and Hispanic/Latino respondents at different values of energy-adjusted AHEI-2010, Fig. 1 plots the estimated slope of the difference in change in cystatin C for White and Hispanic/Latino respondents on the *y* axis, using White respondents as the reference category, across the range of energy-adjusted AHEI-2010 scores on the *x* axis. Below the centred and rescaled energy-adjusted AHEI-2010 values of -1·16, corresponding to a value of 39 on the original energy-adjusted AHEI-2010 scale, Hispanic/Latino individuals had significantly slower increases in cystatin C than White individuals, demonstrated by the negative value of the estimated slope and the lack of overlap between the value of zero and the 95 % CI for the estimate.

**Fig. 1.**
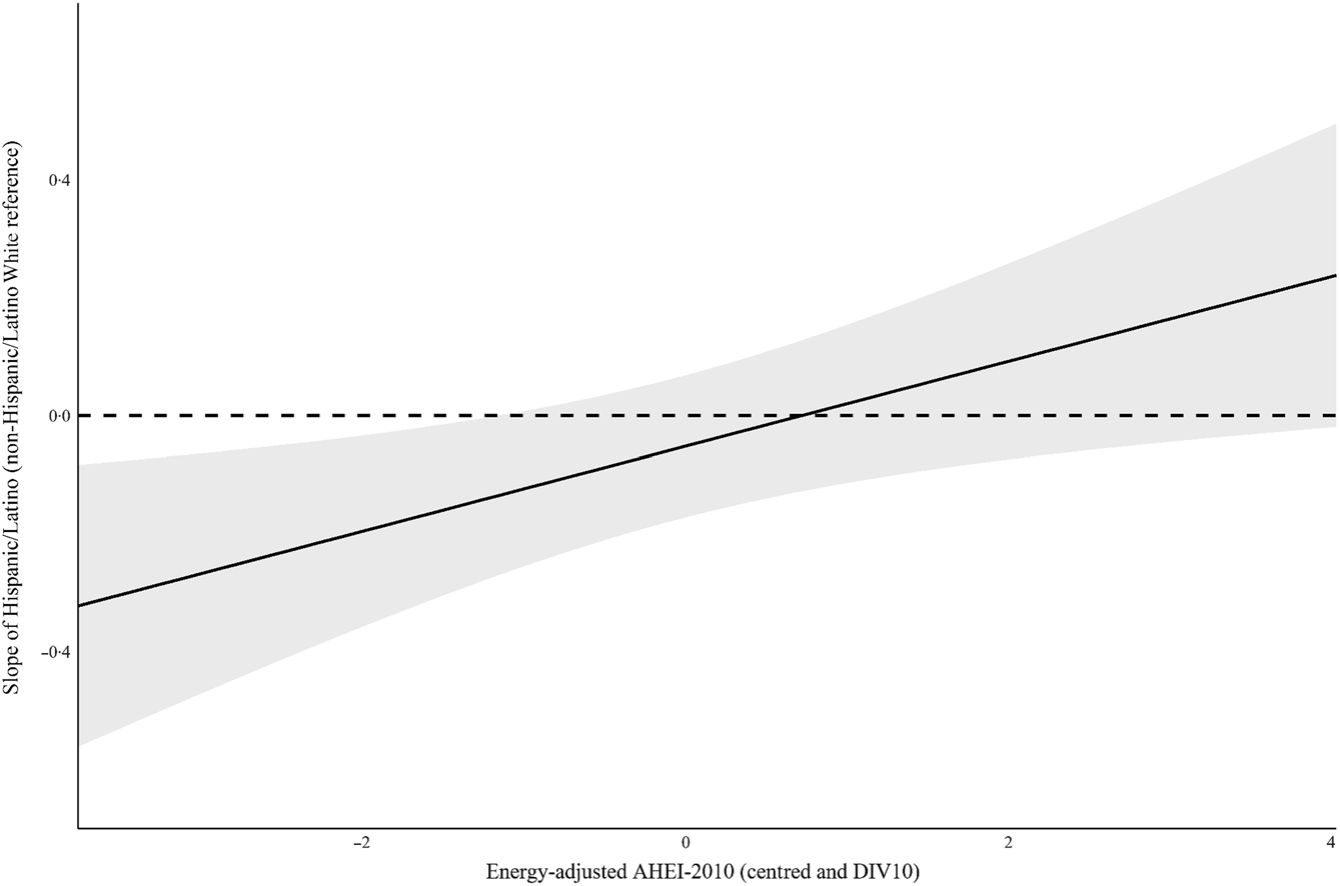
Johnson–Neyman plot of adjusted slope of Hispanic/Latino race/ethnicity, using non-Hispanic/Latino White race/ethnicity as reference category, by energy-adjusted AHEI-2010 (DIV10) when predicting change in cystatin C, 2012–2016. Note: Energy-adjusted AHEI-2010 DIV10: Energy-adjusted Alternative Healthy Eating Index-2010, score divided by 10 before entered as independent variable. Model used to estimate Johnson–Neyman plot included statistical adjustment for baseline cystatin C (2012), age, sex, marital status, retirement status, interview language, nativity, education, household income, household assets, food security status, BMI, vigorous physical activity, smoking status, multimorbidity, diabetic kidney problems, blood pressure, HbA1c, cholesterol, C-reactive protein, and energy intake.

### Supplementary analyses

Supplementary analyses were conducted to provide context for the observed differences in the association between change in cystatin C and energy-adjusted AHEI-2010 scores among White and Hispanic/Latino respondents. Supplementary Table 1 presents results from regression models testing for baseline differences in cystatin C by race/ethnicity. In the baseline direct effect model, there were no significant differences in baseline cystatin C related to respondent race/ethnicity. When interaction terms between energy-adjusted AHEI-2010 and indicator variables for race/ethnicity were included, there was no evidence that the association between energy-adjusted AHEI-2010 and baseline cystatin C differed by racial/ethnic background.

Supplementary Table 2 displays weighted means, standard deviations, and statistical comparisons for non-energy-adjusted AHEI-2010 components, macronutrient density, and select nutrient intake by race/ethnicity. Based on the for non-energy-adjusted AHEI-2010 scored components, Hispanic/Latino respondents had significantly higher scores than White respondents on intake of vegetables, whole grains, and sugar-sweetened beverages but had significantly lower intake of nuts and legumes. Percent of daily energy from carbohydrate and fat did differ by race/ethnicity, with Hispanic/Latino respondents having a greater percentage of daily energy content from carbohydrate and a lower percentage of daily energy content from fat than White respondents. For measures of protein intake, Hispanic/Latino respondents had significantly greater intake of vegetable protein than White respondents. Compared with White respondents, Hispanic/Latino respondents also reported significantly greater intake of vitamin B_6_, food folate, and dietary folate equivalents.

Supplementary Table 3 reports descriptive statistics and results from difference testing for biomarkers related to diabetes and kidney function by race/ethnicity using the additional biomarkers from the 2016 HRS VBS. Compared with White respondents in 2016, Hispanic/Latino respondents had significantly greater blood protein levels, and lower levels of bilirubin, though no other significant differences between White and Hispanic/Latino older adults were identified.

## Discussion

Using data from a weighted sample of ageing Americans, we found that Hispanic/Latino older adults with moderately low to low dietary quality measured using energy-adjusted AHEI-2010 scores experienced less rapid change in cystatin C from 2012 to 2016 than White older adults with comparable dietary quality. To our knowledge, we are the first to demonstrate racial/ethnic differences in the association between dietary quality and longitudinal change in cystatin C among older adults. Our work contributes to the growing body of research identifying healthy dietary patterns to be generally protective of kidney function^(26,29)^, though our findings suggest that dietary quality may be differentially associated with kidney function for older adults from different racial/ethnic backgrounds. At moderately low to low levels of dietary quality, cystatin C tended to increase less rapidly for older Hispanic/Latinos than for older Whites, likely reflecting the role of race/ethnicity in determining dietary choices and possible resiliency to the deleterious effects of low-quality dietary intake in ageing Hispanic/Latino populations. Though demographic and artefactual effects may contribute to the observed results, future research on differential exposure and outcomes of poor dietary quality among diverse groups of older adults is required.

When comparing Hispanic/Latino and White older adults reporting moderately low to low dietary quality, Hispanic/Latino older adults had slower increase in cystatin C than did White older adults, indicating that exposure to lower dietary quality was potentially more harmful to the kidney function of White compared to Hispanic/Latino older adults. These findings align with the Hispanic Paradox theory suggesting Hispanic/Latinos may have better health and mortality outcomes than expected, given their unequal risk of experiencing structural disadvantage^(59,60)^, but it is unclear whether these advantages are rooted in protective cultural mechanisms, the results of selection through immigration and return migration, or are artefactual^(61)^. Further work is necessary to identify the underlying mechanisms that allow Hispanic/Latino older adults to better manage the effects of lower dietary quality on kidney function.

Comparison of several measures of dietary quality across racial/ethnic background provided supporting evidence for our findings. Though there were not overall differences in energy-adjusted AHEI-2010 when comparing White and Hispanic/Latino older adults, Hispanic/Latinos had significantly greater intake of vegetables and whole grains than White older adults, and Hispanic/Latinos reported significantly greater intake of vegetable protein, vitamin B_6_, and folate. Greater vegetable protein intake has been associated with reduced proteinuria and urinary creatinine^(62)^ and reduced mortality among those experiencing CKD^(63)^. Vitamin B_6_ has been shown to inhibit formation of advanced glycation end products^(64)^, and inadequate folate intake has been associated with hyperhomocysteinemia, which is common in CKD patients^(65)^. That Hispanic/Latino older adults were consuming some nephroprotective foods and nutrients in greater quantities than older White adults may underly racial/ethnic differences in the observed association between dietary quality and cystatin C change.

A final consideration is heterogeneity in dietary intake among the Hispanic/Latino older adults related to nativity and acculturation. Hispanic/Latino women born in the USA are known to have inferior dietary intake to their non US born counterparts^(66,67)^, and greater acculturation has been negatively associated with intake of fibre, fruits and vegetables^(68,69)^, plasma concentration of carotenoids^(70)^, and positively associated with percentage of energy from fat^(67)^. As only 37·4 % of Hispanic/Latino older adults included in our analyses reported being born in the USA, and only a small percentage of older White or Black adults were born outside of the USA, the potentially superior dietary intake of immigrant Hispanic/Latino older adults may have contributed to the observed findings. The AHEI-2010 had been shown to have good validity and reproducibility across racial/ethnic subgroups in the USA^(71)^, though we suspect that dietary intake among non-native Hispanic/Latino older adults may be composed of some foods excluded from the FFQ used in the HCNS and the scoring of the AHEI-2010. Testing the equivalence of dietary indices across racial/ethnic subgroups of older adults, particularly older Hispanic/Latinos, is required to eliminate measurement error as an alternative explanation to our findings.

Though our research benefits from several strengths including longitudinal measurement of cystatin C in a racially/ethnically diverse sample of community-dwelling older Americans, several factors limit the impact of our findings. A primary limitation is the cross-sectional measurement of dietary quality in the HCNS. Variation in dietary quality may be an important factor in changes in cystatin C, other biomarkers, and chronic conditions generally, meaning lack of longitudinal data on dietary intake allows potential alternative explanations of the observed findings. As mentioned, measurement error related to dietary quality in Hispanic/Latino older adults in the HCNS may have contributed to our primary findings. Though based on a previously validated semi-quantitative FFQ^(44)^ that has shown validity in modified forms in a biracial sample of older adults^(46)^, the specific FFQ included in the HRS/HCNS was not been validated on the racially/ethnically diverse sample of older adults included in our study. This may have contributed to bias in estimates of dietary intake and racial/ethnic variation in the association between dietary quality and change in cystatin C, especially among Hispanic/Latinos that were not included in the previously referenced validation study. Another limitation was that disaggregation of Hispanic/Latino subgroups is not possible given the measurement of race/ethnicity in the HRS. Dietary intake has been shown to vary within Hispanic/Latino older adults from different origins^(72)^, and future work should consider heterogeneity of dietary intake within racial/ethnic subgroups. Additionally, unknown or unmeasured confounders cannot be ruled out owing to the nature of observational studies. Finally, about 36·4 % of the age-eligible sample was removed from analyses due to exclusion criteria related to missing follow-up measures on cystatin C, implausible energy intake, and respondents who reported race/ethnicity other than White, Black, or Hispanic/Latino. As older adults with the greatest increases in cystatin C may have been more likely to dropout of the study due to an increased risk of mortality, our analytic sample included only relatively healthy older adults who survived over the 4-year observational period. This may have resulted in conservative estimates of the association between dietary quality, race/ethnicity, and change in cystatin C, so future work using shorter observational periods may be necessary to better represent older adults at the greatest risk of rapid change in cystatin C. Though exclusion based on implausible energy intakes likely provided more reliable estimates of the associations of interest, exclusion of respondents who identify as racial/ethnic minorities including Asian and Native American/American Indian limits the generalisability of our results.

In conclusion, efforts to identify modifiable dietary inputs to the progression of kidney function and CKD in older adults should consider race/ethnicity as a measure that may impact the efficacy of interventions. Programmes that support healthy dietary intake should be considered by clinicians and policy-makers as a means of effectively reducing the burden of kidney disfunction in ageing populations, though racial/ethnic background of programme participants may be an important characteristic influencing the outcomes of such interventions. Future research in this area should also work to ensure valid and reproducible measurement of dietary quality in racial/ethnic and immigrant subgroups.

## Supplementary material

For supplementary material accompanying this paper visit https://doi.org/10.1017/S0007114522001040. click here to view supplementary material
