# Supplementary material for: A prospective cohort study of racial/ethnic variation in the association between change in cystatin C and dietary quality in older Americans
Source: Br J Nutr. 2022 Apr 11;129(2):312–23. doi: 10.1017/S0007114522001040 (PMC9870715; doi:10.1017/S0007114522001040)
Supplement: Supplementary file 1 [file S0007114522001040sup001.docx]

| **Supplementary Table 1.** Linear regression estimates of the association between baseline cystatin C (2012), energy adjusted AHEI-2010, and race/ethnicity; Health and Retirement Study (2012) and Health Care and Nutrition Study (2013) | | | | | | | |
| --- | --- | --- | --- | --- | --- | --- | --- |
|  | Baseline direct effect model | | | Baseline interaction model, Non-Hispanic/Latino White reference | | | |
| *Primary Measures* | *b* | 95% CI | | *b* | 95% CI | |  |
| Energy-adjusted AHEI-2010 (DIV10) | -0.03 | (-0.05, | -0.01) | -0.03 | (-0.05, | -0.01) |  |
| Race/ethnicity |  |  |  |  |  |  |  |
| Non-Hispanic/Latino White (ref) | -0.03 | (-0.14, | 0.08) | -0.04 | (-0.15, | 0.06) |  |
| Non-Hispanic/Latino Black | 0.14 | (-0.10, | 0.39) | 0.15 | (-0.10, | 0.39) |  |
| Hispanic/Latino |  |  |  |  |  |  |  |
| *Interaction Terms* |  |  |  |  |  |  |  |
| Energy-adjusted AHEI-2010* Non-Hispanic/Latino Black |  |  |  | -0.04 | (-0.14, | 0.06) |  |
| Energy-adjusted AHEI-2010* Hispanic/Latino |  |  |  | 0.01 | (-0.06, | 0.09) |  |
| *Covariates* |  |  |  |  |  |  |  |
| Age | 0.02 | (0.01, | 0.02) | 0.02 | (0.01, | 0.02) |  |
| Female | -0.02 | (-0.07, | 0.04) | -0.02 | (-0.07, | 0.03) |  |
| Married/partnered | -0.01 | (-0.08, | 0.06) | -0.01 | (-0.08, | 0.06) |  |
| Retired | 0.03 | (-0.02, | 0.08) | 0.03 | (-0.02, | 0.08) |  |
| Spanish interview | -0.21 | (-0.47, | 0.05) | -0.22 | (-0.47, | 0.03) |  |
| Native born | 0.11 | (0.03, | 0.20) | 0.12 | (0.04, | 0.20) |  |
| Education |  |  |  |  |  |  |  |
| < HS degree | 0.10 | (0.00, | 0.20) | 0.1 | (0.00, | 0.20) |  |
| HS degree (reference) |  |  |  |  |  |  |  |
| > HS degree | -0.02 | (-0.08, | 0.03) | -0.02 | (-0.08, | 0.04) |  |
| Household assets, log | -0.01 | (-0.01, | 0.00) | -0.01 | (-0.01, | 0.00) |  |
| Household income, log | -0.02 | (-0.06, | 0.01) | -0.02 | (-0.06, | 0.01) |  |
| Food insecure | -0.06 | (-0.17, | 0.04) | -0.06 | (-0.17, | 0.04) |  |
| Body Mass Index |  |  |  |  |  |  |  |
| Underweight | 0.04 | (-0.26, | 0.34) | 0.04 | (-0.26, | 0.34) |  |
| Normal (reference) |  |  |  |  |  |  |  |
| Overweight | -0.01 | (-0.08, | 0.07) | -0.01 | (-0.08, | 0.07) |  |
| Obese | 0.07 | (-0.01, | 0.15) | 0.07 | (-0.01, | 0.15) |  |
| Vigorous Physical Activity |  |  |  |  |  |  |  |
| None (reference) |  |  |  |  |  |  |  |
| Moderate | -0.04 | (-0.10, | 0.03) | -0.04 | (-0.10, | 0.03) |  |
| Regular | -0.05 | (-0.11, | 0.01) | -0.06 | (-0.11, | 0.00) |  |
| Current smoker | 0.05 | (-0.05, | 0.15) | 0.05 | (-0.05, | 0.16) |  |
| Sum chronic conditions | 0.01 | (-0.01, | 0.04) | 0.01 | (-0.01, | 0.04) |  |
| Diabetic kidney problems | 0.57 | (0.19, | 0.95) | 0.58 | (0.20, | 0.96) |  |
| Blood Pressure |  |  |  |  |  |  |  |
| Normal (reference) |  |  |  |  |  |  |  |
| Elevated | -0.08 | (-0.17, | 0.01) | -0.08 | (-0.17, | 0.00) |  |
| Stage 1 hypertensive | -0.08 | (-0.16, | 0.01) | -0.08 | (-0.16, | 0.01) |  |
| Stage 2 hypertensive | -0.13 | (-0.21, | -0.05) | -0.13 | (-0.21, | -0.05) |  |
| Hypertensive crisis | -0.03 | (-0.23, | 0.17) | -0.03 | (-0.23, | 0.18) |  |
| Hypotensive | -0.02 | (-0.21, | 0.17) | -0.02 | (-0.21, | 0.17) |  |
| HbA1c |  |  |  |  |  |  |  |
| Normal (reference) |  |  |  |  |  |  |  |
| Pre-diabetic | 0.05 | (-0.01, | 0.11) | 0.05 | (-0.01, | 0.11) |  |
| Diabetic | 0.12 | (0.02, | 0.22) | 0.12 | (0.02, | 0.22) |  |
| Cholesterol |  |  |  |  |  |  |  |
| Desirable (reference) |  |  |  |  |  |  |  |
| Borderline high | -0.01 | (-0.06, | 0.05) | -0.01 | (-0.06, | 0.05) |  |
| High | 0.10 | (0.03, | 0.18) | 0.1 | (0.03, | 0.18) |  |
| C-reactive protein (log) | 0.09 | (0.05, | 0.13) | 0.09 | (0.05, | 0.13) |  |
| Energy intake (Kcal/100) | 0.00 | (0.00, | 0.01) | 0 | (0.00, | 0.01) |  |
| R squared | 0.25 | | | 0.26 | | | |
| Energy-adjusted AHEI-2010 (DIV 10): Energy-adjusted Alternative Healthy Eating Index-2010, divided by 10.  HS: high school.  *b*: regression estimate; 95% CI: 95% confidence interval.  Estimates adjusted for complex sample design. | | | | | | | |

| **Supplementary Table 2.** AHEI-2010 components, energy intake, macronutrient density, and select nutrient intake by race/ethnicity, Health Care and Nutrition Study (2013) | | | | | | | | | | | | | | | | | | | | | | | | | | | |
| --- | --- | --- | --- | --- | --- | --- | --- | --- | --- | --- | --- | --- | --- | --- | --- | --- | --- | --- | --- | --- | --- | --- | --- | --- | --- | --- | --- |
| *AHEI-2010 Scored Component^‡^* | Total Sample | | | | Non-Hispanic/  Latino White | | | | Non-Hispanic/  Latino Black | | | | | | | Hispanic/Latino | | | | | | |  | | | |  |
|  | N | Mean | SD | N | | Mean | | SD | N | | Mean | | | SD | | N | | Mean | | | SD | | | F | | *p* | |
| Vegetables, servings/d | 958 | 5.0 | 2.6 | 789 | | 4.9 | ^a^ | 2.5 | 108 | 4.4 | | ^a^ | 2.6 | | 61 | | 6.7 | |  | 2.9 | | 12.3 | | | <0.001 | | |
| Fruit, servings/d | 958 | 3.9 | 2.4 | 789 | | 3.9 |  | 2.4 | 108 | 3.6 | |  | 2.3 | | 61 | | 4.4 | |  | 2.8 | | 1.5 | | | 0.233 | | |
| Whole grain, g/d | 958 | 3.5 | 2.6 | 789 | | 3.4 | ^a^ | 2.4 | 108 | 3.5 | | ^a^ | 2.6 | | 61 | | 5.7 | |  | 3.4 | | 16.9 | | | <0.001 | | |
| Sugar-sweetened beverages, servings/d | 958 | 4.4 | 38 | 789 | | 4.6 | ^a^ | 3.8 | 108 | 2.5 | | ^b^ | 3.3 | | 61 | | 3.8 | | ^a,b,^ | 3.8 | | 10.8 | | | <0.001 | | |
| Nuts and legumes, servings/d | 958 | 6.4 | 3.2 | 789 | | 6.5 | ^a^ | 3.2 | 108 | 5.0 | | ^b^ | 3.2 | | 61 | | 5.9 | | ^a,b^ | 3.1 | | 7.7 | | | <0.001 | | |
| Red/processed meats, servings/d | 958 | 5.0 | 2.7 | 789 | | 5.0 |  | 2.7 | 108 | 4.8 | |  | 3.1 | | 61 | | 5.9 | |  | 2.3 | | 2.4 | | | 0.089 | | |
| Trans fat, % of energy | 958 | 8.5 | 0.8 | 789 | | 8.5 |  | 0.8 | 108 | 8.5 | |  | 0.8 | | 61 | | 8.7 | |  | 0.8 | | 1.9 | | | 0.146 | | |
| Long-chain (n-3) fats (EPA + DHA), mg/d | 958 | 4.2 | 2.9 | 789 | | 4.1 |  | 2.9 | 108 | 5.0 | |  | 3.0 | | 61 | | 4.2 | |  | 3.2 | | 2.7 | | | 0.065 | | |
| PUFA, % of energy | 958 | 6.2 | 2.1 | 789 | | 6.2 |  | 2.1 | 108 | 5.8 | |  | 1.9 | | 61 | | 5.8 | |  | 1.8 | | 1.6 | | | 0.206 | | |
| Alcohol, drinks/d | 958 | 5.6 | 3.1 | 789 | | 5.7 | ^a,b^ | 3.1 | 108 | 4.9 | | ^a,c^ | 2.8 | | 61 | | 4.9 | | ^b,c^ | 2.7 | | 3.2 | | | 0.043 | | |
| Sodium, mg/d | 958 | 5.4 | 2.9 | 789 | | 5.4 | ^a,b^ | 2.8 | 108 | 6.3 | | ^a^ | 3.2 | | 61 | | 4.6 | | ^b^ | 3.1 | | 4.9 | | | 0.007 | | |
| *Macronutrient Density* |  |  |  |  | |  |  |  |  |  | |  |  | |  | |  | |  |  | |  | | |  | | |
| % Carbohydrate | 958 | 52.1 | 8.5 | 789 | | 51.7 |  | 8.3 | 108 | 55.0 | | ^a^ | 9.0 | | 61 | | 55.4 | | ^a^ | 7.8 | | 7.9 | | | <0.001 | | |
| % Protein | 958 | 16.6 | 3.2 | 789 | | 16.5 |  | 3.2 | 108 | 17.0 | |  | 3.4 | | 61 | | 17.1 | |  | 3.1 | | 1.17 | | | 0.309 | | |
| % Fat | 958 | 35.1 | 6.6 | 789 | | 35.3 | ^a^ | 6.5 | 108 | 33.3 | | ^a,b^ | 7.1 | | 61 | | 33.2 | | ^b^ | 6.4 | | 4.72 | | | 0.009 | | |
| *Nutrient Intake* |  |  |  |  | |  |  |  |  |  | |  |  | |  | |  | |  |  | |  | | |  | | |
| Protein, gm | 958 | 68.8 | 27.3 | 789 | | 68.5 |  | 26.2 | 108 | 67.6 | |  | 32.8 | | 61 | | 75.4 | |  | 29.8 | | 1.5 | | | 0.230 | | |
| Animal protein, gm | 958 | 43.8 | 20.3 | 789 | | 43.6 |  | 19.3 | 108 | 45.6 | |  | 25.9 | | 61 | | 45.2 | |  | 20.7 | | 0.4 | | | 0.663 | | |
| Vegetable protein, gm | 958 | 24.9 | 12.1 | 789 | | 24.9 |  | 12.3 | 108 | 22.1 | | ^a^ | 10.1 | | 61 | | 30.2 | | ^a^ | 13.1 | | 6.3 | | | 0.002 | | |
| Zinc, mg | 958 | 10.8 | 4.3 | 789 | | 10.9 |  | 4.2 | 108 | 9.9 | |  | 4.9 | | 61 | | 11.4 | |  | 4.8 | | 2.1 | | | 0.127 | | |
| Riboflavin, mg | 958 | 2.0 | 1.2 | 789 | | 2.0 |  | 1.2 | 108 | 1.8 | |  | 0.9 | | 61 | | 2.1 | |  | 1.0 | | 1.1 | | | 0.349 | | |
| Vitamin B6, (pyridoxine), mg | 958 | 1.8 | 0.8 | 789 | | 1.8 |  | 0.7 | 108 | 1.7 | | ^a^ | 1.0 | | 61 | | 2.1 | | ^a^ | 0.9 | | 4.0 | | | 0.019 | | |
| Vitamin B12, mcg | 958 | 6.2 | 3.5 | 789 | | 6.1 | ^a,b^ | 3.2 | 108 | 7.1 | | ^a,c^ | 4.6 | | 61 | | 6.4 | | ^b,c^ | 3.8 | | 3.1 | | | 0.044 | | |
| Food folate, mcg | 958 | 262.2 | 119.9 | 789 | | 259.6 | ^a^ | 114.6 | 108 | 239.2 | | ^a^ | 120.3 | | 61 | | 346.0 | |  | 167.9 | | 12.5 | | | <.001 | | |
| Folic acid, mcg | 958 | 147.2 | 103.9 | 789 | | 147.8 |  | 98.0 | 108 | 131.7 | |  | 135.6 | | 61 | | 159.2 | |  | 114.1 | | 1.2 | | | 0.300 | | |
| Dietary folate equivalents, mcg | 958 | 512.4 | 234.9 | 789 | | 510.9 | ^a^ | 219.7 | 108 | 463.1 | | ^a^ | 294.9 | | 61 | | 616.7 | |  | 290.4 | | 6.4 | | | 0.002 | | |
| Lutein and zeaxanthin, mcg | 958 | 2408.5 | 2182.2 | 789 | | 2351.9 | ^a^ | 2130.1 | 108 | 2549.3 | |  | 2408.6 | | 61 | | 3275.3 | | ^a^ | 2377.6 | | 4.0 | | | 0.019 | | |
| Total trans fat, g | 958 | 1.7 | 0.8 | 789 | | 1.7 |  | 0.8 | 108 | 1.6 | |  | 1.0 | | 61 | | 1.6 | |  | 0.7 | | 0.6 | | | 0.563 | | |
| Total omega 3, g | 958 | 1.5 | 0.9 | 789 | | 1.5 |  | 0.9 | 108 | 1.4 | |  | 0.9 | | 61 | | 1.6 | |  | 0.9 | | 0.7 | | | 0.509 | | |
| Total omega 6, g | 958 | 11.8 | 6.0 | 789 | | 11.7 |  | 6.0 | 108 | 10.9 | |  | 6.4 | | 61 | | 11.7 | |  | 5.3 | | 0.8 | | | 0.447 | | |
| Methionine, gm | 958 | 1.5 | 0.6 | 789 | | 1.5 |  | 0.6 | 108 | 1.5 | |  | 0.8 | | 61 | | 1.6 | |  | 0.7 | | 1.3 | | | 0.265 | | |
| Free choline, choline-contributing metabolite, mg | 958 | 74.0 | 30.2 | 789 | | 74.0 | ^a^ | 29.1 | 108 | 64.0 | |  | 29.9 | | 61 | | 88.5 | | ^a^ | 41.9 | | 9.0 | | | <0.001 | | |
| Choline from phosphocholine, mg | 958 | 12.9 | 6.5 | 789 | | 12.6 | ^a,b^ | 6.0 | 108 | 12.1 | | ^a,c^ | 6.6 | | 61 | | 18.4 | | ^b,c^ | 10.8 | | 16.5 | | | <0.001 | | |
| Choline from phosphatidylcholine, mg | 958 | 157.7 | 75.7 | 789 | | 157.2 |  | 73.7 | 108 | 157.6 | |  | 88.2 | | 61 | | 168.7 | |  | 76.1 | | 0.5 | | | 0.608 | | |
| Total Choline (no betaine), mg | 958 | 309.6 | 123.3 | 789 | | 308.8 |  | 118.6 | 108 | 295.5 | |  | 142.7 | | 61 | | 345.4 | |  | 146.4 | | 2.4 | | | 0.093 | | |
| Betaine, choline derivative, mg | 958 | 83.2 | 42.3 | 789 | | 83.9 |  | 42.0 | 108 | 78.6 | |  | 46.1 | | 61 | | 77.5 | |  | 38.2 | | 0.9 | | | 0.398 | | |
| AHEI-2010: Alternative Healthy Eating Index-2010; SD: standard deviation.  ^a,b,c^ Matching superscript letters denote non-significant pairwise comparison.  *^‡^* AHEI-2010 Scored Component (0 =worst, 10 = best). Scoring criteria described elsewhere.^30^  ^+^ significantly greater observed versus expected count, ^-^ significantly fewer observed versus expected count in cell.  Overall differences in continuous measures were compared using ANOVA, with least squares mean differences used for bivariate follow-up.  The significance level for each follow-up test was adjusted using the Bonferroni correction.  Means and statistical tests account for complex sample design. | | | | | | | | | | | | | | | | | | | | | | | | | | | |

| **Supplementary Table 3.** Select biomarkers related to kidney function and diabetes by race/ethnicity, Health and Retirement Study Venous Blood Study (2016) | | | | | | | | | | | | | | | | | | | | | | | | | | | |
| --- | --- | --- | --- | --- | --- | --- | --- | --- | --- | --- | --- | --- | --- | --- | --- | --- | --- | --- | --- | --- | --- | --- | --- | --- | --- | --- | --- |
|  | Total Sample | | | | Non-Hispanic/  Latino White | | | | Non-Hispanic/  Latino Black | | | | | | | Hispanic/Latino | | | | | | |  | | | |  |
|  | N | Mean | SD | N | | Mean | | SD | N | | Mean | | | SD | | N | | Mean | | | SD | | | F | | *p* | |
| Creatinine, mg/dL | 733 | 1.0 | 0.4 | 614 | | 1.0 | ^a,b^ | 0.3 | 78 | 1.2 | | ^a,c^ | 0.8 | | 41 | | 1.0 | | ^b,c^ | 0.4 | | 10.47 | | | <0.001 | | |
| Blood protein, g/dL | 733 | 6.8 | 0.4 | 614 | | 6.8 |  | 0.4 | 78 | 7.0 | | ^a^ | 0.5 | | 41 | | 7.0 | | ^a^ | 0.4 | | 11.71 | | | <0.001 | | |
| Albumin, g/dL | 733 | 3.9 | 0.3 | 614 | | 3.9 | ^a^ | 0.3 | 78 | 3.8 | | ^b^ | 0.4 | | 41 | | 3.9 | | ^a,b^ | 0.2 | | 6.66 | | | 0.001 | | |
| Alanine aminotransferase, u/L | 732 | 18.7 | 11.0 | 613 | | 18.9 |  | 8.7 | 78 | 17.1 | |  | 22.6 | | 41 | | 18.2 | |  | 10.2 | | 0.76 | | | 0.466 | | |
| Aspartate aminotransferase, u/L | 729 | 21.8 | 9.2 | 610 | | 21.8 |  | 7.6 | 78 | 22.0 | |  | 17.5 | | 41 | | 21.1 | |  | 8.1 | | 0.12 | | | 0.891 | | |
| Bilirubin (total), mg/dL | 733 | 0.5 | 0.3 | 614 | | 0.5 |  | 0.3 | 78 | 0.3 | |  | 0.2 | | 41 | | 0.4 | |  | 0.2 | | 9.13 | | | <0.001 | | |
| Blood urea nitrogen - mg/dL | 733 | 19.7 | 8.0 | 614 | | 19.7 |  | 7.9 | 78 | 19.7 | |  | 9.5 | | 41 | | 18.6 | |  | 7.8 | | 0.29 | | | 0.745 | | |
| LDL-C (calculated), mg/dL | 729 | 95.7 | 34.2 | 611 | | 95.7 |  | 34.4 | 78 | 101.3 | |  | 35.6 | | 40 | | 87.7 | |  | 26.4 | | 1.46 | | | 0.232 | | |
| Triglycerides, mg/dL | 733 | 132.6 | 66.3 | 614 | | 131.9 |  | 65.0 | 78 | 124.8 | |  | 59.0 | | 41 | | 162.0 | |  | 85.2 | | 3.31 | | | 0.037 | | |
| SD: standard deviation; LDL-C: low density lipoprotein cholesterol.  ^a,b,c^ Matching superscript letters denote non-significant pairwise comparison.  ^+^ significantly greater observed versus expected count, ^-^ significantly fewer observed versus expected count in cell.  Means and statistical tests account for complex sample design. | | | | | | | | | | | | | | | | | | | | | | | | | | | |


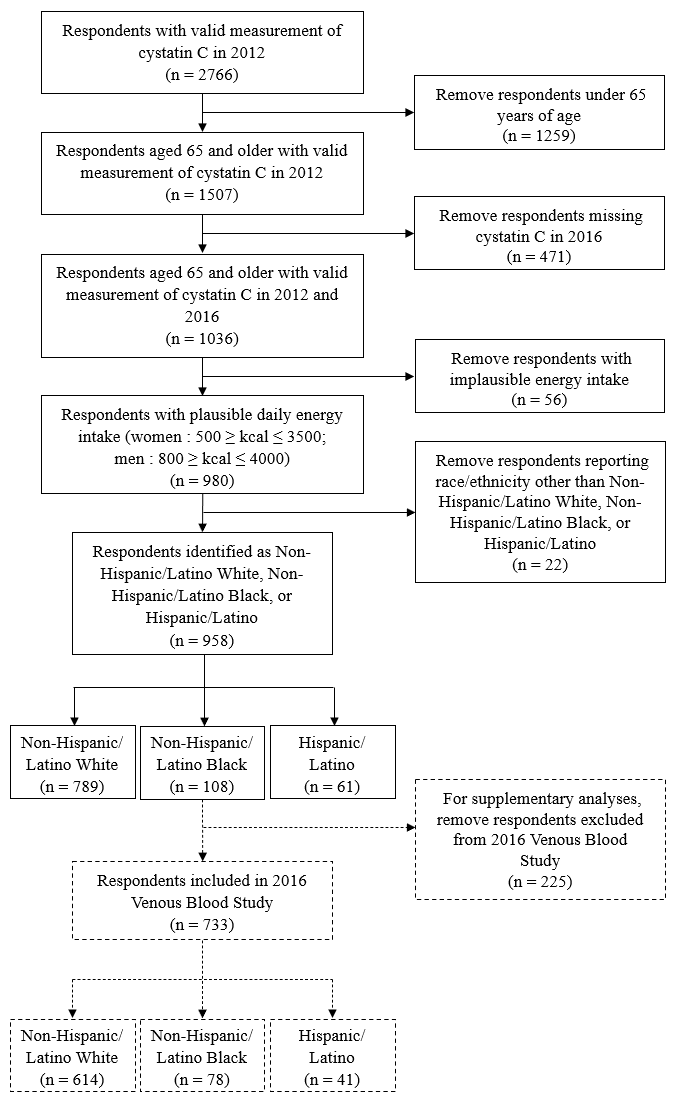


**Supplementary Figure 1.** Participant Selection Flowchart

Note: Dashed lines represent sub-sample used for secondary analyses.
